# Bis{2-[3-(dimethyl­amino)propyl­imino­meth­yl]-4,6-dihydro­seleno­phenolato}zinc(II)

**DOI:** 10.1107/S1600536809032061

**Published:** 2009-08-19

**Authors:** Jun Yang, Ji-Wen Yuan, Ru-Hua Zha, Qing-Fu Zeng

**Affiliations:** a Engineering Research Center for Clean Production of Textile Dyeing and Printing, Ministry of Education, Wuhan 430073, People’s Republic of China

## Abstract

In the title complex, [Zn(C_12_H_17_N_2_OSe_2_)_2_], the Zn^II^ ion is six-coordinated by two *N*,*N*′,*O*-tridentate Schiff base ligands, resulting in a slightly distorted *trans*-ZnO_2_N_4_ octa­hedral coordination for the metal ion.

## Related literature

For background to Schiff bases as ligands, see: Shi *et al.* (2008 ▶); Xu *et al.* (2009 ▶). For reference structural data see: Allen *et al.* (1987 ▶).
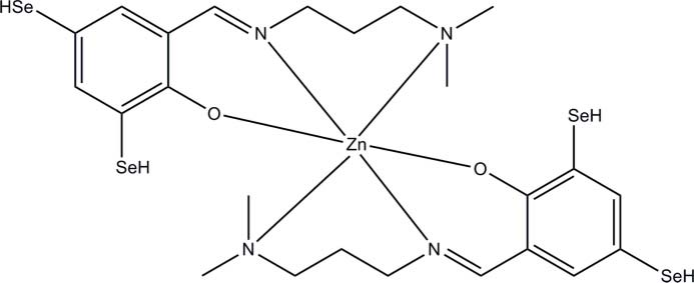


## Experimental

### 

#### Crystal data

[Zn(C_12_H_17_N_2_OSe_2_)_2_]
*M* *_r_* = 791.76
Orthorhombic, 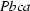

*a* = 15.8066 (16) Å
*b* = 16.875 (3) Å
*c* = 21.297 (3) Å
*V* = 5680.4 (13) Å^3^
*Z* = 8
Mo *K*α radiation
μ = 6.02 mm^−1^
*T* = 296 K
0.35 × 0.28 × 0.24 mm

#### Data collection

Enraf–Nonius CAD-4 diffractometer
Absorption correction: ψ scan (North *et al.*, 1968 ▶) *T*_min_ = 0.227, *T*_max_ = 0.326 (expected range = 0.164–0.236)
28494 measured reflections
5005 independent reflections
3434 reflections with *I* > 2σ(*I*)
*R*_int_ = 0.064
3 standard reflections every 200 reflections intensity decay: 1%

#### Refinement

*R*[*F* ^2^ > 2σ(*F* ^2^)] = 0.041
*wR*(*F* ^2^) = 0.117
*S* = 1.03
5005 reflections
324 parameters
H-atom parameters constrained
Δρ_max_ = 0.66 e Å^−3^
Δρ_min_ = −1.41 e Å^−3^

### 

Data collection: *CAD-4 Software* (Enraf–Nonius, 1989 ▶); cell refinement: *CAD-4 Software*; data reduction: *XCAD4* (Harms & Wocadlo, 1995 ▶); program(s) used to solve structure: *SHELXS97* (Sheldrick, 2008 ▶); program(s) used to refine structure: *SHELXL97* (Sheldrick, 2008 ▶); molecular graphics: *SHELXTL* (Sheldrick, 2008 ▶); software used to prepare material for publication: *SHELXTL*.

## Supplementary Material

Crystal structure: contains datablocks global, I. DOI: 10.1107/S1600536809032061/hb5046sup1.cif

Structure factors: contains datablocks I. DOI: 10.1107/S1600536809032061/hb5046Isup2.hkl

Additional supplementary materials:  crystallographic information; 3D view; checkCIF report

## Figures and Tables

**Table 1:** Selected bond lengths (Å)

Zn1—N1	2.096 (5)
Zn1—N2	2.092 (5)
Zn1—N3	2.330 (5)
Zn1—N4	2.362 (5)
Zn1—O1	2.031 (4)
Zn1—O3	2.017 (4)

**Fig. 1.**
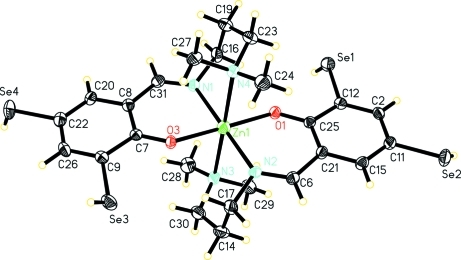
The molecular structure of (I) showing 30% probability displacement ellipsoids.

## supplementary crystallographic information

### Comment

There has been much research interest in Schiff base metal complexes due to their molecular architectures and biological activities (Shi *et al.*, 2008; Xu *et al.*, 2009). In this work, we report here the crystal structure of the title compound, (I). In (I), all bond lengths are within normal ranges (Allen *et al.*, 1987) (Fig. 1). The Zn(II) is six-coordinated by two O and four N atoms from the two Schiff base ligands, forming a slightly distorted octahedral coordination (Table 1, Fig. 1).

### Experimental

A mixture of 3,5-dihydroseleno-2-hydroxybenzaldehyde (564 mg, 2 mmol), *N*,*N*-dimethylpropane-1,3-diamine (204 mg, 2 mmol) and ZnCl2 (1 mmol, 134 mg) in methanol (10 ml) was stirred for 1 h. After keeping the filtrate in air for 7 d, colourless blocks of (I) were formed.

### Refinement

All H atoms were positioned geometrically (C—H = 0.93–0.96Å, Se—H = 0.82Å) and refined as riding, with *U*_iso_(H) = 1.2*U*_eq_(carrier) or 1.5*U*_eq_(methyl C).

### Crystal data

**Table:**

[Zn(C_12_H_17_N_2_OSe_2_)_2_]	*F*(000) = 3104
*M**_r_* = 791.76	*D*_x_ = 1.852 Mg m^−^^3^
Orthorhombic, *P**b**c**a*	Mo *K*α radiation, λ = 0.71073 Å
Hall symbol: -P 2ac 2ab	Cell parameters from 25 reflections
*a* = 15.8066 (16) Å	θ = 9–12°
*b* = 16.875 (3) Å	µ = 6.02 mm^−^^1^
*c* = 21.297 (3) Å	*T* = 296 K
*V* = 5680.4 (13) Å^3^	Block, colourless
*Z* = 8	0.35 × 0.28 × 0.24 mm

### Data collection

**Table:**

Enraf–Nonius CAD-4 diffractometer	3434 reflections with *I* > 2σ(*I*)
Radiation source: fine-focus sealed tube	*R*_int_ = 0.064
graphite	θ_max_ = 25.0°, θ_min_ = 1.9°
ω/2θ scans	*h* = −17→18
Absorption correction: ψ scan (North *et al.*, 1968)	*k* = −19→20
*T*_min_ = 0.227, *T*_max_ = 0.326	*l* = −23→25
28494 measured reflections	3 standard reflections every 200 reflections
5005 independent reflections	intensity decay: 1%

### Refinement

**Table:**

Refinement on *F*^2^	Primary atom site location: structure-invariant direct methods
Least-squares matrix: full	Secondary atom site location: difference Fourier map
*R*[*F*^2^ > 2σ(*F*^2^)] = 0.041	Hydrogen site location: inferred from neighbouring sites
*wR*(*F*^2^) = 0.117	H-atom parameters constrained
*S* = 1.03	*w* = 1/[σ^2^(*F*_o_^2^) + (0.0534*P*)^2^ + 15.0374*P*] where *P* = (*F*_o_^2^ + 2*F*_c_^2^)/3
5005 reflections	(Δ/σ)_max_ = 0.001
324 parameters	Δρ_max_ = 0.66 e Å^−^^3^
0 restraints	Δρ_min_ = −1.41 e Å^−^^3^

### Special details

**Table:**

Geometry. All e.s.d.'s (except the e.s.d. in the dihedral angle between two l.s. planes) are estimated using the full covariance matrix. The cell e.s.d.'s are taken into account individually in the estimation of e.s.d.'s in distances, angles and torsion angles; correlations between e.s.d.'s in cell parameters are only used when they are defined by crystal symmetry. An approximate (isotropic) treatment of cell e.s.d.'s is used for estimating e.s.d.'s involving l.s. planes.
Refinement. Refinement of *F*^2^ against ALL reflections. The weighted *R*-factor *wR* and goodness of fit *S* are based on *F*^2^, conventional *R*-factors *R* are based on *F*, with *F* set to zero for negative *F*^2^. The threshold expression of *F*^2^ > σ(*F*^2^) is used only for calculating *R*-factors(gt) *etc*. and is not relevant to the choice of reflections for refinement. *R*-factors based on *F*^2^ are statistically about twice as large as those based on *F*, and *R*- factors based on ALL data will be even larger.

### Fractional atomic coordinates and isotropic or equivalent isotropic displacement parameters (Å^2^)

**Table:**

	*x*	*y*	*z*	*U*_iso_*/*U*_eq_	
C2	0.3561 (4)	0.3166 (3)	0.8608 (3)	0.0317 (14)	
H2	0.3380	0.3435	0.8964	0.038*	
C6	0.4433 (4)	0.1993 (4)	0.6968 (3)	0.0337 (14)	
H6	0.4983	0.2131	0.6856	0.040*	
C7	0.2377 (4)	−0.0020 (3)	0.5639 (2)	0.0259 (13)	
C8	0.1506 (4)	−0.0082 (3)	0.5798 (3)	0.0308 (14)	
C9	0.2599 (4)	−0.0376 (3)	0.5056 (3)	0.0305 (14)	
C11	0.4385 (4)	0.3222 (3)	0.8414 (3)	0.0339 (14)	
C12	0.3006 (4)	0.2705 (3)	0.8270 (3)	0.0303 (14)	
C14	0.4154 (5)	0.1968 (4)	0.5531 (3)	0.0493 (19)	
H14A	0.4286	0.2492	0.5690	0.059*	
H14B	0.4471	0.1897	0.5146	0.059*	
C15	0.4655 (4)	0.2818 (3)	0.7888 (3)	0.0345 (14)	
H15	0.5217	0.2854	0.7762	0.041*	
C16	0.1130 (4)	0.0867 (4)	0.7353 (3)	0.0429 (17)	
H16A	0.0521	0.0834	0.7302	0.052*	
H16B	0.1269	0.1396	0.7498	0.052*	
C17	0.4470 (4)	0.1364 (4)	0.6005 (3)	0.0409 (16)	
H17A	0.4344	0.0832	0.5860	0.049*	
H17B	0.5078	0.1412	0.6053	0.049*	
C19	0.1420 (5)	0.0261 (4)	0.7831 (3)	0.0517 (19)	
H19A	0.1094	0.0338	0.8212	0.062*	
H19B	0.1284	−0.0262	0.7672	0.062*	
C20	0.0937 (4)	−0.0493 (3)	0.5409 (3)	0.0336 (14)	
H20	0.0371	−0.0534	0.5523	0.040*	
C21	0.4089 (4)	0.2357 (3)	0.7544 (3)	0.0305 (13)	
C22	0.1210 (4)	−0.0829 (4)	0.4868 (3)	0.0374 (15)	
C23	0.2340 (5)	0.0274 (4)	0.8000 (3)	0.0460 (17)	
H23A	0.2468	0.0784	0.8188	0.055*	
H23B	0.2440	−0.0128	0.8317	0.055*	
C24	0.3801 (5)	0.0185 (4)	0.7721 (3)	0.0508 (18)	
H24A	0.3868	−0.0196	0.8052	0.076*	
H24B	0.3902	0.0707	0.7882	0.076*	
H24C	0.4199	0.0073	0.7392	0.076*	
C25	0.3231 (4)	0.2284 (3)	0.7711 (3)	0.0283 (13)	
C26	0.2039 (4)	−0.0770 (4)	0.4687 (3)	0.0364 (15)	
H26	0.2218	−0.0998	0.4312	0.044*	
C27	0.2824 (4)	−0.0665 (3)	0.7214 (3)	0.0411 (16)	
H27A	0.2894	−0.1048	0.7543	0.062*	
H27B	0.3235	−0.0759	0.6891	0.062*	
H27C	0.2266	−0.0711	0.7040	0.062*	
C28	0.1761 (5)	0.2009 (4)	0.5649 (3)	0.0516 (19)	
H28A	0.1671	0.2424	0.5348	0.077*	
H28B	0.1363	0.2062	0.5986	0.077*	
H28C	0.1685	0.1504	0.5448	0.077*	
C29	0.2727 (5)	0.2868 (4)	0.6164 (3)	0.0471 (18)	
H29A	0.2673	0.3253	0.5835	0.071*	
H29B	0.3275	0.2915	0.6356	0.071*	
H29C	0.2297	0.2957	0.6475	0.071*	
C30	0.3234 (5)	0.1949 (4)	0.5373 (3)	0.0463 (18)	
H30A	0.3126	0.2356	0.5062	0.056*	
H30B	0.3112	0.1442	0.5178	0.056*	
C31	0.1173 (4)	0.0247 (3)	0.6373 (3)	0.0333 (14)	
H31	0.0624	0.0100	0.6480	0.040*	
N1	0.1546 (3)	0.0715 (3)	0.6751 (2)	0.0295 (11)	
N2	0.4049 (3)	0.1510 (3)	0.6611 (2)	0.0309 (11)	
N3	0.2629 (3)	0.2064 (3)	0.5899 (2)	0.0353 (12)	
N4	0.2942 (3)	0.0139 (3)	0.7472 (2)	0.0341 (12)	
O1	0.2677 (3)	0.1893 (2)	0.74031 (17)	0.0326 (9)	
O3	0.2936 (2)	0.0318 (2)	0.59826 (17)	0.0292 (9)	
Se1	0.18790 (4)	0.26148 (5)	0.85594 (3)	0.0481 (2)	
H1A	0.1555	0.2789	0.8292	0.072*	
Se2	0.51657 (5)	0.38473 (4)	0.88781 (3)	0.0482 (2)	
H2A	0.4928	0.4248	0.9006	0.072*	
Se3	0.37374 (5)	−0.02917 (5)	0.47921 (3)	0.0479 (2)	
H3A	0.3906	−0.0727	0.4675	0.072*	
Se4	0.04414 (5)	−0.14005 (4)	0.43431 (3)	0.0517 (2)	
H4A	0.0342	−0.1141	0.4026	0.078*	
Zn1	0.27994 (5)	0.11131 (4)	0.66822 (3)	0.0408 (2)	

### Atomic displacement parameters (Å^2^)

**Table:**

	*U*^11^	*U*^22^	*U*^33^	*U*^12^	*U*^13^	*U*^23^
C2	0.038 (4)	0.032 (3)	0.025 (3)	0.000 (3)	0.001 (3)	−0.003 (3)
C6	0.029 (4)	0.038 (4)	0.035 (3)	−0.002 (3)	0.001 (3)	−0.002 (3)
C7	0.031 (4)	0.021 (3)	0.026 (3)	0.004 (3)	−0.001 (3)	0.005 (2)
C8	0.034 (4)	0.027 (3)	0.031 (3)	0.002 (3)	−0.002 (3)	0.000 (3)
C9	0.037 (4)	0.025 (3)	0.029 (3)	0.007 (3)	0.001 (3)	0.004 (2)
C11	0.038 (4)	0.029 (3)	0.035 (3)	−0.006 (3)	−0.002 (3)	−0.001 (3)
C12	0.032 (3)	0.029 (3)	0.030 (3)	0.003 (3)	0.003 (3)	0.001 (3)
C14	0.061 (5)	0.048 (4)	0.038 (4)	−0.007 (4)	0.019 (4)	−0.001 (3)
C15	0.026 (3)	0.038 (3)	0.040 (3)	−0.002 (3)	0.001 (3)	−0.002 (3)
C16	0.027 (4)	0.061 (4)	0.041 (4)	−0.007 (3)	0.009 (3)	−0.023 (3)
C17	0.031 (4)	0.051 (4)	0.040 (4)	0.001 (3)	0.012 (3)	−0.017 (3)
C19	0.062 (5)	0.060 (5)	0.033 (4)	−0.013 (4)	0.015 (3)	−0.004 (3)
C20	0.029 (4)	0.032 (3)	0.040 (3)	−0.003 (3)	−0.003 (3)	−0.004 (3)
C21	0.026 (3)	0.033 (3)	0.033 (3)	−0.002 (3)	−0.001 (3)	−0.005 (3)
C22	0.043 (4)	0.031 (3)	0.038 (4)	0.001 (3)	−0.009 (3)	0.000 (3)
C23	0.061 (5)	0.047 (4)	0.030 (3)	−0.006 (4)	0.002 (3)	0.006 (3)
C24	0.052 (5)	0.050 (4)	0.050 (4)	−0.004 (4)	−0.007 (4)	0.010 (3)
C25	0.030 (3)	0.025 (3)	0.030 (3)	−0.002 (3)	−0.001 (3)	0.001 (3)
C26	0.052 (4)	0.032 (3)	0.025 (3)	0.008 (3)	−0.001 (3)	−0.004 (3)
C27	0.052 (4)	0.027 (3)	0.044 (4)	−0.003 (3)	0.002 (3)	0.006 (3)
C28	0.064 (5)	0.038 (4)	0.053 (4)	0.008 (4)	−0.005 (4)	0.005 (3)
C29	0.069 (5)	0.033 (4)	0.039 (4)	0.006 (3)	0.005 (4)	0.002 (3)
C30	0.064 (5)	0.041 (4)	0.034 (4)	−0.001 (4)	0.008 (3)	0.001 (3)
C31	0.026 (3)	0.034 (3)	0.041 (3)	0.000 (3)	0.001 (3)	0.001 (3)
N1	0.021 (3)	0.035 (3)	0.033 (3)	−0.002 (2)	0.003 (2)	−0.005 (2)
N2	0.023 (3)	0.037 (3)	0.032 (3)	−0.003 (2)	0.007 (2)	−0.004 (2)
N3	0.040 (3)	0.029 (3)	0.037 (3)	0.000 (2)	−0.001 (2)	−0.001 (2)
N4	0.038 (3)	0.031 (3)	0.033 (3)	−0.005 (2)	−0.001 (2)	−0.001 (2)
O1	0.030 (2)	0.035 (2)	0.033 (2)	−0.0030 (19)	0.0047 (19)	−0.0088 (19)
O3	0.026 (2)	0.032 (2)	0.029 (2)	−0.0031 (18)	−0.0007 (17)	−0.0074 (18)
Se1	0.0359 (4)	0.0637 (5)	0.0448 (4)	−0.0061 (4)	0.0120 (3)	−0.0130 (4)
Se2	0.0418 (4)	0.0535 (5)	0.0492 (4)	−0.0096 (3)	−0.0053 (3)	−0.0182 (3)
Se3	0.0401 (4)	0.0656 (5)	0.0379 (4)	0.0013 (4)	0.0104 (3)	−0.0119 (3)
Se4	0.0552 (5)	0.0488 (4)	0.0511 (4)	0.0001 (4)	−0.0231 (4)	−0.0151 (3)
Zn1	0.0388 (5)	0.0432 (5)	0.0404 (4)	−0.0027 (4)	0.0028 (4)	−0.0059 (3)

### Geometric parameters (Å, °)

**Table:**

C2—C11	1.370 (8)	C22—Se4	1.912 (6)
C2—C12	1.376 (8)	C23—N4	1.491 (8)
C2—H2	0.9300	C23—H23A	0.9700
C6—N2	1.269 (7)	C23—H23B	0.9700
C6—C21	1.475 (8)	C24—N4	1.461 (8)
C6—H6	0.9300	C24—H24A	0.9600
C7—O3	1.280 (7)	C24—H24B	0.9600
C7—C8	1.421 (8)	C24—H24C	0.9600
C7—C9	1.423 (8)	C25—O1	1.277 (6)
C8—C20	1.406 (8)	C26—H26	0.9300
C8—C31	1.446 (8)	C27—N4	1.476 (7)
C9—C26	1.359 (8)	C27—H27A	0.9600
C9—Se3	1.890 (6)	C27—H27B	0.9600
C11—C15	1.378 (8)	C27—H27C	0.9600
C11—Se2	1.901 (6)	C28—N3	1.474 (9)
C12—C25	1.432 (8)	C28—H28A	0.9600
C12—Se1	1.892 (6)	C28—H28B	0.9600
C14—C30	1.492 (10)	C28—H28C	0.9600
C14—C17	1.518 (9)	C29—N3	1.477 (8)
C14—H14A	0.9700	C29—H29A	0.9600
C14—H14B	0.9700	C29—H29B	0.9600
C15—C21	1.394 (8)	C29—H29C	0.9600
C15—H15	0.9300	C30—N3	1.486 (8)
C16—N1	1.463 (7)	C30—H30A	0.9700
C16—C19	1.514 (9)	C30—H30B	0.9700
C16—H16A	0.9700	C31—N1	1.273 (7)
C16—H16B	0.9700	C31—H31	0.9300
C17—N2	1.472 (7)	Zn1—N1	2.096 (5)
C17—H17A	0.9700	Zn1—N2	2.092 (5)
C17—H17B	0.9700	Zn1—N3	2.330 (5)
C19—C23	1.499 (10)	Zn1—N4	2.362 (5)
C19—H19A	0.9700	Zn1—O1	2.031 (4)
C19—H19B	0.9700	Zn1—O3	2.017 (4)
C20—C22	1.354 (8)	Se1—H1A	0.8200
C20—H20	0.9300	Se2—H2A	0.8200
C21—C25	1.407 (8)	Se3—H3A	0.8200
C22—C26	1.370 (9)	Se4—H4A	0.8200
			
C11—C2—C12	119.1 (5)	C21—C25—C12	113.9 (5)
C11—C2—H2	120.4	C9—C26—C22	119.7 (6)
C12—C2—H2	120.4	C9—C26—H26	120.1
N2—C6—C21	126.1 (6)	C22—C26—H26	120.1
N2—C6—H6	117.0	N4—C27—H27A	109.5
C21—C6—H6	117.0	N4—C27—H27B	109.5
O3—C7—C8	124.4 (5)	H27A—C27—H27B	109.5
O3—C7—C9	121.1 (5)	N4—C27—H27C	109.5
C8—C7—C9	114.5 (5)	H27A—C27—H27C	109.5
C20—C8—C7	121.1 (5)	H27B—C27—H27C	109.5
C20—C8—C31	117.1 (6)	N3—C28—H28A	109.5
C7—C8—C31	121.8 (5)	N3—C28—H28B	109.5
C26—C9—C7	123.5 (6)	H28A—C28—H28B	109.5
C26—C9—Se3	119.0 (5)	N3—C28—H28C	109.5
C7—C9—Se3	117.6 (4)	H28A—C28—H28C	109.5
C2—C11—C15	120.3 (6)	H28B—C28—H28C	109.5
C2—C11—Se2	119.9 (4)	N3—C29—H29A	109.5
C15—C11—Se2	119.8 (5)	N3—C29—H29B	109.5
C2—C12—C25	123.9 (6)	H29A—C29—H29B	109.5
C2—C12—Se1	118.4 (4)	N3—C29—H29C	109.5
C25—C12—Se1	117.7 (4)	H29A—C29—H29C	109.5
C30—C14—C17	117.2 (6)	H29B—C29—H29C	109.5
C30—C14—H14A	108.0	N3—C30—C14	117.0 (5)
C17—C14—H14A	108.0	N3—C30—H30A	108.0
C30—C14—H14B	108.0	C14—C30—H30A	108.0
C17—C14—H14B	108.0	N3—C30—H30B	108.0
H14A—C14—H14B	107.3	C14—C30—H30B	108.0
C11—C15—C21	120.4 (6)	H30A—C30—H30B	107.3
C11—C15—H15	119.8	N1—C31—C8	127.2 (6)
C21—C15—H15	119.8	N1—C31—H31	116.4
N1—C16—C19	109.6 (5)	C8—C31—H31	116.4
N1—C16—H16A	109.8	C31—N1—C16	116.9 (5)
C19—C16—H16A	109.8	C31—N1—Zn1	126.4 (4)
N1—C16—H16B	109.8	C16—N1—Zn1	115.5 (4)
C19—C16—H16B	109.8	C6—N2—C17	114.6 (5)
H16A—C16—H16B	108.2	C6—N2—Zn1	127.8 (4)
N2—C17—C14	108.7 (5)	C17—N2—Zn1	115.9 (4)
N2—C17—H17A	110.0	C28—N3—C29	107.1 (5)
C14—C17—H17A	110.0	C28—N3—C30	108.5 (5)
N2—C17—H17B	110.0	C29—N3—C30	109.9 (5)
C14—C17—H17B	110.0	C28—N3—Zn1	108.9 (4)
H17A—C17—H17B	108.3	C29—N3—Zn1	110.3 (4)
C23—C19—C16	116.4 (6)	C30—N3—Zn1	112.0 (4)
C23—C19—H19A	108.2	C24—N4—C27	107.5 (5)
C16—C19—H19A	108.2	C24—N4—C23	108.1 (5)
C23—C19—H19B	108.2	C27—N4—C23	109.9 (5)
C16—C19—H19B	108.2	C24—N4—Zn1	108.1 (4)
H19A—C19—H19B	107.3	C27—N4—Zn1	111.3 (4)
C22—C20—C8	120.2 (6)	C23—N4—Zn1	111.7 (4)
C22—C20—H20	119.9	C25—O1—Zn1	131.0 (4)
C8—C20—H20	119.9	C7—O3—Zn1	130.1 (4)
C15—C21—C25	122.3 (5)	C12—Se1—H1A	109.5
C15—C21—C6	115.7 (5)	C11—Se2—H2A	109.5
C25—C21—C6	121.9 (5)	C9—Se3—H3A	109.5
C20—C22—C26	120.9 (6)	C22—Se4—H4A	109.5
C20—C22—Se4	120.4 (5)	O3—Zn1—O1	178.46 (16)
C26—C22—Se4	118.7 (5)	O3—Zn1—N2	93.35 (17)
N4—C23—C19	115.8 (5)	O1—Zn1—N2	86.43 (17)
N4—C23—H23A	108.3	O3—Zn1—N1	86.55 (17)
C19—C23—H23A	108.3	O1—Zn1—N1	93.68 (17)
N4—C23—H23B	108.3	N2—Zn1—N1	179.8 (2)
C19—C23—H23B	108.3	O3—Zn1—N3	86.68 (16)
H23A—C23—H23B	107.4	O1—Zn1—N3	94.79 (16)
N4—C24—H24A	109.5	N2—Zn1—N3	80.60 (19)
N4—C24—H24B	109.5	N1—Zn1—N3	99.28 (19)
H24A—C24—H24B	109.5	O3—Zn1—N4	93.03 (16)
N4—C24—H24C	109.5	O1—Zn1—N4	85.51 (16)
H24A—C24—H24C	109.5	N2—Zn1—N4	100.64 (18)
H24B—C24—H24C	109.5	N1—Zn1—N4	79.48 (18)
O1—C25—C21	125.2 (5)	N3—Zn1—N4	178.74 (18)
O1—C25—C12	120.9 (5)		
